# ﻿New nematode *Tahaminaindica* gen. nov., sp. nov. (Nematoda, Dorylaimida, Tylencholaimoidea) from the tropical rainforest, Western Ghats

**DOI:** 10.3897/zookeys.1186.101527

**Published:** 2023-12-06

**Authors:** Md Niraul Islam, Wasim Ahmad

**Affiliations:** 1 Institute of Applied Ecology, Chinese Academy of Sciences, Shenyang-110016, China Aligarh Muslim University Aligarh India; 2 Nematode Biodiversity Research Lab, Department of Zoology, Aligarh Muslim University, Aligarh-202002, India Institute of Applied Ecology, Chinese Academy of Sciences Shenyang China

**Keywords:** Morphometrics, new genus, new species, SEM, taxonomy

## Abstract

During a nematological survey in the Western Ghats a new nematode belonging to the superfamily Tylencholaimoida (Dorylaimida) extracted from the rhizosphere of the soil of grasses, is described and illustrated. *Tahaminaindica***gen. nov.**, **sp. nov.** is characterized by females with a body length of 1.3–1.4 mm; lip region 8.0 μm wide, approximately one-fourth of the body diameter at the pharyngeal base; amphidial fovea cup-shaped, about one-half as wide as the lip region diameter. Odontostyle 8.0–9.0 μm long, 1.0–1.1 times lip region diameter; guiding ring simple; odontophore rod-like, 10.5–11.5 μm long with basal thickening or minute knobs-like structure; pharynx consisting of a weakly muscular anterior part, expanding abruptly into a cylindrical basal bulb, occupying about two-fifths to one-half of the total pharyngeal length; female genital system monodelphic-opisthodelphic with anterior uterine sac; vulval opening pore-like, and tail elongated with a slight dorsally curved tip. Males not found.

## ﻿Introduction

The Western Ghats is a fascinating biogeographic region located in southern India, and is one of 36 world biodiversity hotspots (Myers 1988; Myers et al. 2000). According to the latest biodiversity survey, the Western Ghats represents about 30% of India’s biodiversity, with a significant number of endemic species (CEPF 2016) and is home to 325 threatened species. The average temperature varies from 20 °C in the south to 24 °C in the north, and because of precipitation in some areas, it dips below freezing during winter. The main types of habitats include forests (shola, moist deciduous, dry deciduous, evergreen, semi-evergreen), bamboo breaks, savanna and rocky areas. Rainfall on the Western Ghats’s Mountain slopes ranges from 1000 to 7600 mm annually, with an average of 2500 mm (Gadgil 1994, 1996a, 1996b). This is a highly mosaic biogeographic area with rich biodiversity and great environmental variation in topography, soil type, rainfall, and temperature (Gadgil 1996b). Many different invertebrate species are known from this hotspot, including thousands of arthropods, annelids and hundreds of molluscs. However, records of nematofauna from the Western Ghats are scant. Jairajpuri and Ahmad (1982) and Ahmad and Jairajpuri (1985) described two new nematode genera *Aporcedorus* and *Silvallis* respectively from this hotspot. In a series of papers, Ahmad and Jairajpuri (1982–1988), Ahmad et al. (1992), Ahmad and Ahmad (1992), Ahmad and Ahmad (2002), Ahmad (1993) and Dhanam and Jairajpuri (1999) have recorded several species of soil-inhabiting nematodes from this region (Islam and Ahmad 2021a). Recently, Islam and Ahmad (2021–2022) and Islam et al. (2019) in their publications described several new and known species of tylencholaimoid nematodes from the Western Ghats.

In this study, large numbers of soil samples were collected from several localities of the Western Ghats. These samples yielded several species of the superfamily Tylencholaimoidea (Dorylaimida), with most published recently by Islam and Ahmad (l.c.). One sample also contained a nematode taxon with a morphology that is distinct from any known genus. Given the documented morphological distinctiveness, a new genus and species is described in detail herein.

## ﻿Material and methods

### ﻿Soil sampling and nematode extraction and processing

Soil samples were collected from the rhizosphere of grasses (*Cynodondactylon* L.) from Yaraganalu, Shivamoga District, Karnataka State, India. The nematodes were extracted from soil by using Cobb’s (1918) sieving and decantation and modified Baermann’s funnel methods. Then the specimens were fixed in hot triethanolamine-glycerol fixative and transferred to a glycerine-alcohol solution in a small cavity block which was then kept in a desiccator containing anhydrous calcium chloride for slow dehydration and mounted in anhydrous glycerin. The paraffin wax ring method was used to make permanent mounts (De Maeseneer and d’Herde 1963).

### ﻿Light microscopy study

An ocular micrometer was used to take measurements, a drawing tube was used to make line drawings, and a Nikon DS digital camera connected to a Nikon Eclipse 80i microscope was used to capture photographs.

### ﻿Scanning Electron Microscopy (SEM) study

The nematodes were fixated in 4% formaldehyde, transferred to distilled water for 2 hours and then dehydrated in a graded ethanol-acetone series (25% for 24 hours and 25, 30, 50, 70, 80, 90, 95 and 100% for 2 hours each step) and followed by acetone (100% for 1 hour). Following dehydration, samples were dried using critical point drying (Leica-EM CPD 300), coated with a gold sputter (Jeol-JFC 1600), and then observed with a Jeol-JSM 6510 LV SEM.

## ﻿Taxonomy

### ﻿ Tahamina gen. nov.

Taxon classificationAnimaliaDorylaimidaTylencholaimoidea

9DB4F2FD-F9E1-501D-A922-C2B883AC9ACB

https://zoobank.org/66077C36-1977-4004-89BF-B2AE34774B4B

Tylencholaiminae Filipjev, 1934 (Tylencholaimidae Filipjev, 1934).

#### Diagnosis.

Large sized nematode, 1.3–1.4 mm long body. Cuticle dorylaimoid, outer cuticle thin with very fine transverse striation, inner layer thicker than the outer, distinctly striated. Lateral chords narrow. Lip region cap-like, offset by constriction, 8.0 μm wide or one-fourth of the body diameter at pharyngeal base, labial papillae raised, lips slightly separated. Cheilostom a truncate cone. Amphidial fovea cup-shaped, aperture slit-like, occupying about one-half as wide as lip region diameter. Odontostyle short, robust, spindle-shaped, 8.0–9.0 μm long, 1.0–1.1 times lip region diameter. Odontophore simple, rod-like, 10.5–11.5 μm long with thickening or minute knobs-like structure at the base. Guiding ring simple. Pharynx consists of a slender and weakly muscular anterior part expanding abruptly into a cylindrical basal bulb, separated by constriction, occupying about two-fifths to one-half (42–50%) of the total pharyngeal length. Female genital system monodelphic-opisthodelphic with anterior uterine sac. Vulva pore-like. Tail long, elongated with a slight dorsally curved tip. Male not known.

#### Type and only species.

*Tahaminaindica* gen. nov., sp. nov.

#### Relationships.

In the presence of a short odontostyle with a distinct lumen, odontophore with minute knobs-like structure and longer pharyngeal expansion, the new genus well fits under the subfamily Tylencholaiminae Filipjev, 1934 of the family Tylencholaimidae (Tylencholaimoidea).

*Tahaminaindica* gen. nov. can be separated from *Tylencholaimus* de Man, 1876 in having a dorylaimoid cuticle, radial elements absent (vs. tylencholaimoid cuticle, radial elements present); vulva pore-like (vs. vulva transverse); differently shaped tail (tail long, elongated vs. tail short, rounded to conoid). The new genus differs from the *Heynsnema* Peňa-Santiago, Guerrero & Ciobanu, 2008 in having female genital system mono-opisthodelphic (vs. amphidelphic); vulva pore-like (vs. vulva transverse) and differently shaped tail (tail long, elongated vs. tail short, rounded to conoid).

Based on the shape and size of the odontostyle and odontophore, the expanded part of the pharynx and tail, the new genus comes close to *Discomyctus* Thorne, 1939 and *Wasimellus* Bloemers & Wanless, 1996 but differs from the former in having the inner cuticle dorylaimoid (vs. tylencholaimoid); labial disc absent and labial papillae raised (vs. labial disc present and labial papillae not raised); odontophore with thickened base or minute basal knobs-like structure (vs. odontophore with distinct large basal knobs); female genital system mono-opisthodelphic (vs. mono-prodelphic); vulva pore-like (vs. transverse) and tail elongated (vs. tail elongated to tail long filiform). It differs from *Wasimellus* in having a guiding ring single (vs. guiding ring double), the expanded part of the pharynx comparatively long (42–49 vs. 35–42% of total neck length), pharyngeal expansion abrupt (vs. gradual expansion); vulva pore-like without sclerotized pieces (vs. vulva transverse, with sclerotized pieces); female genital system mono-opisthodelphic (vs. amphidelphic), tail elongated (vs. tail filiform).

In the presence of a dorylaimoid cuticle, narrow lateral chord, and weakly muscular expanded part of the pharynx, the new genus resembles *Dorylaimoides* Thorne & Swanger, 1936, which belongs to the family Mydonomidae but differs in having a differently shaped odontostyle (odontostyle spindle-shaped, symmetrical vs. odontostyle simple, asymmetrical) and odontophore (odontophore simple rod-like with basal thickening or very minute knobs-like structure vs. odontophore angular or arcuate without basal thickening or knobs); longer pharyngeal expansion (about two-fifths to one-half vs. one-fifth to one-third of total neck length); and vulva pore-like (vs. vulva transverse).

Based on the shape of the stylet, lip region and tail pattern, the new genus is comparable to two non-tylencholaimid members *Mitoaxonchium* Yeates, 1973 and *Hulqus* Siddiqi, 1981, both belonging to the subfamily Hulqinae of the family Qudsianematidae (Dorylaimoidea) but it differs from the former in having a longer body size (L = 1.3–1.4 vs. 0.46–0.61 mm); shorter pharynx (b = 4.9–5.2 vs. 2.4–3.2); the position of dorsal esophageal gland nuclei more anteriorly (12–16 vs. 30% of expanded part of pharynx from its expansion), female genital system mono-opisthodelphic (vs. amphidelphic) and differently shaped vulval opening (pore-like vs. transverse). The new genus differs from *Hulqus* in having a longer body (L = 1.3–1.4 vs. 0.86–1.10 mm); shorter pharynx (b = 4.9–5.2 vs. 2.4–3.0); the position of dorsal esophageal gland nuclei more anteriorly (12–16 vs. 35–41% of expanded part of pharynx from its expansion), pharyngeal expansion abrupt, separated by constriction (vs. expansion gradual, without constriction) and differently shaped vulval opening (pore-like vs. transverse).

### ﻿ Tahamina indica sp. nov.

Taxon classificationAnimaliaDorylaimidaTylencholaimoidea

D017E00D-B4FD-52E4-AA29-92E3476575D8

https://zoobank.org/D76FB896-7ADD-4B6A-9529-3FE31353E335

Figs 1 , 2 , 3 , 4

#### Material examined.

Seven females and two juveniles from Yaraganalu, Shivamoga District, Karnataka State, India; 13°48'04.3"N 75°34'23.4"E; 17 November 2018. Female ***holotype*** (slide number AMUZDNC *Tahaminaindica* gen. nov. sp. nov. /1), six female ***paratype*** specimens (slide numbers AMUZDNC *Tahaminaindica* gen. nov. sp. nov. /2–5) and two juveniles (J2) (slide number AMUZDNC *Tahaminaindica* gen. nov. sp. nov. /6) are deposited with the nematode collection in the Department of Zoology, Aligarh Muslim University, India.

#### Measurements.

See Table 1.

**Table 1.** Morphometrics of *Tahaminaindica* gen. n*ov.*, sp. nov. All measurements are in μm and in the form: mean ± SD (range).

Characters	Holotype female	Paratype females	Juveniles
n	1	6	2
L	1413	1379.8±34.9 (1332–1430)	1022, 1077
Body diameter at neck base	27	28.1±0.68 (27–29)	21.5, 22.5
Body diameter at mid body	30	31±0.48 (30–31)	21.5, 22.5
Body diameter at anus	16.5	17.3±0.44 (16.5–17.5)	15.0, 15.5
a	46.5	43.8±1.2 (43.1–46.7)	47.4, 47.8
b	5.2	5.01±0.10 (4.9–5.2)	4.2, 4.4
c	15.3	15.2±1.0 (13.7–17.0)	12.9, 13.7
c’	5.5	5.0±0.42 (4.5–5.8)	5.4, 5.0
V	39.5	39.2±1.1 (38.3–41.9)	–
G1	4.9	3.1±0.82 (2.4–3.4)	–
G2	13.8	12.6±0.90 (11.5–14.2)	–
Lip region diameter	8.0	8.0	7.5, 7.0
Lip region height	4.0	3.7±0.24 (3.5–4.0)	3.5, 3.0
Amphid aperture	5.0	4.4±0.43 (4.0–5.0)	4.0, 4.0
Odontostyle length	8.5	8.4±0.31 (8.0–9.0)	7.0, 6.5
Replacement odontostyle length	–	–	8.0, 7.5
Odontophore length	10.5	10.7±0.31 (10.5–11.5)	11, 10.5
Total stylet length	19	19.1±0.45 (19–20)	18, 17.5
Guiding ring from anterior	6.0	5.7±0.18 (5.5–6.0)	5.5, 5.5
Nerve ring from anterior	93	93.2±0.62 (92–94)	83, 90
Neck length	271	270.2±4.9 (259–276)	243, 246
Expanded part of pharynx	127	125.8±4.7 (115–131)	110, 115
Cardia length	6.0	6.4±0.88 (5.0–7.0)	7.5, 6.0
Anterior genital branch	69	40.1±4.4 (34–48)	–
Posterior genital branch	195	178.7±10.1 (168–195)	–
Vaginal length	14.0	14.4±0.44 (13.5–14.5)	–
Vulva from anterior	558	553.5±13.2 (539–579)	–
Prerectum length	157	156.6±13.0 (129–176)	121, 139
Rectum length	20.5	21.5±1.2 (19–23)	18.5, 19.5
Tail length	92	89.3±9.3 (79–103)	79, 78

#### Description.

**Figure 1.**
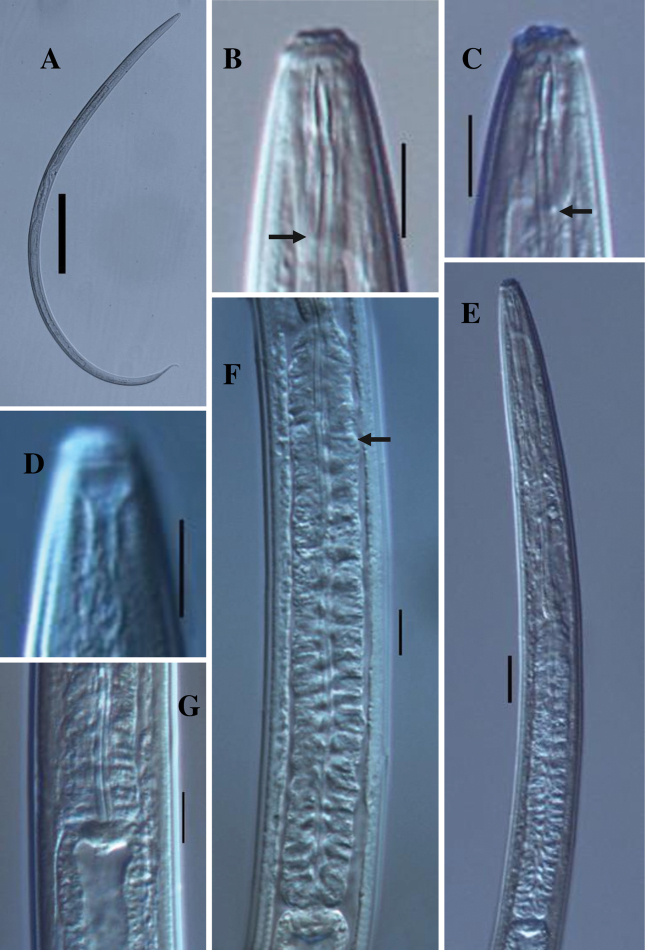
*Tahaminaindica* gen. nov., sp. nov. female: **A** entire **B, C** anterior region (arrow showing knobs-like structure) **D** anterior region showing amphid **E** pharyngeal region **F** expanded part of pharynx (arrow showing dorsal pharyngeal gland nuclei) **G** pharyngo-intestinal junction. Scale bars: 100 µm (**A**); 10 µm (**B–D, F–G**); 20 µm (**E**).

***Female***. Slender nematodes of large sized, 1.3–1.4 mm long body, slightly curved ventrally upon fixation; body cylindrical, tapering gradually towards at the anterior end and posteriorly narrowing to form an elongated tail. Cuticle with two distinct layers, 1.0–1.5 μm thick at anterior region, 2.0–2.5 μm at midbody, and 3.0–4.0 μm on the tail. The outer cuticle is thin, with fine transverse striation, the inner layer is thicker than outer, distinctly striated. Lateral chord 5.0–7.0 μm at midbody, occupying about one-sixth to one-four (18–24%) of corresponding body diameter. Lateral, dorsal and ventral body pores are indistinct. Lip region cap-like, offset from the body by constriction, 2.0–2.2 times as wide as high or about one-fourth of the body diameter at the pharyngeal base. Under SEM (Fig. 3A), lips are rounded, and slightly separated, inner portion somewhat amalgamated forming a hexagonal disk-like structure, offset from the rest of the lip region; labial and cephalic papillae button-like, slightly protruding over the lip surface. Amphidial fovea cup-shaped, aperture slit-like, 4.0–5.0 μm wide or occupying about one-half to three-fifths of lip region diameter. Stoma a truncate cone. Odontostyle short, robust, spindle-shaped with a wide lumen, 1.0–1.1 times the lip region diameter long or 0.54–0.63% of total body length, it’s aperture about one-fourth of the odontostyle length. Odontophore simple, rod-like with slightly thickened base or minute knobs-like structure (Fig. 1B, C), 1.2–1.4 times the odontostyle length. Guiding ring simple refractive, at 0.7–0.8 times lip region diameter from anterior end. Pharynx consisting of a slender and weakly muscular anterior part, expanding abruptly into a cylindroid bulb, separated by slight constriction, occupying about 42–49% of total pharyngeal length. Dorsal pharyngeal gland nuclei located at 12–16% of expanded part of pharynx from its expansion. Pharyngeal gland nuclei and their orifices are located as follows: DO=58–60, DN=60–62, DO–DN=1.5–2.5, S1N1=74–76, S1N2=79–81, S2N2=88–90, S2O=90–91. Nerve ring at 34–35% of the pharyngeal length from anterior end. Cardia is short, conoid, about one-fifth to one-fourth of the corresponding body diameter long.

**Figure 2.**
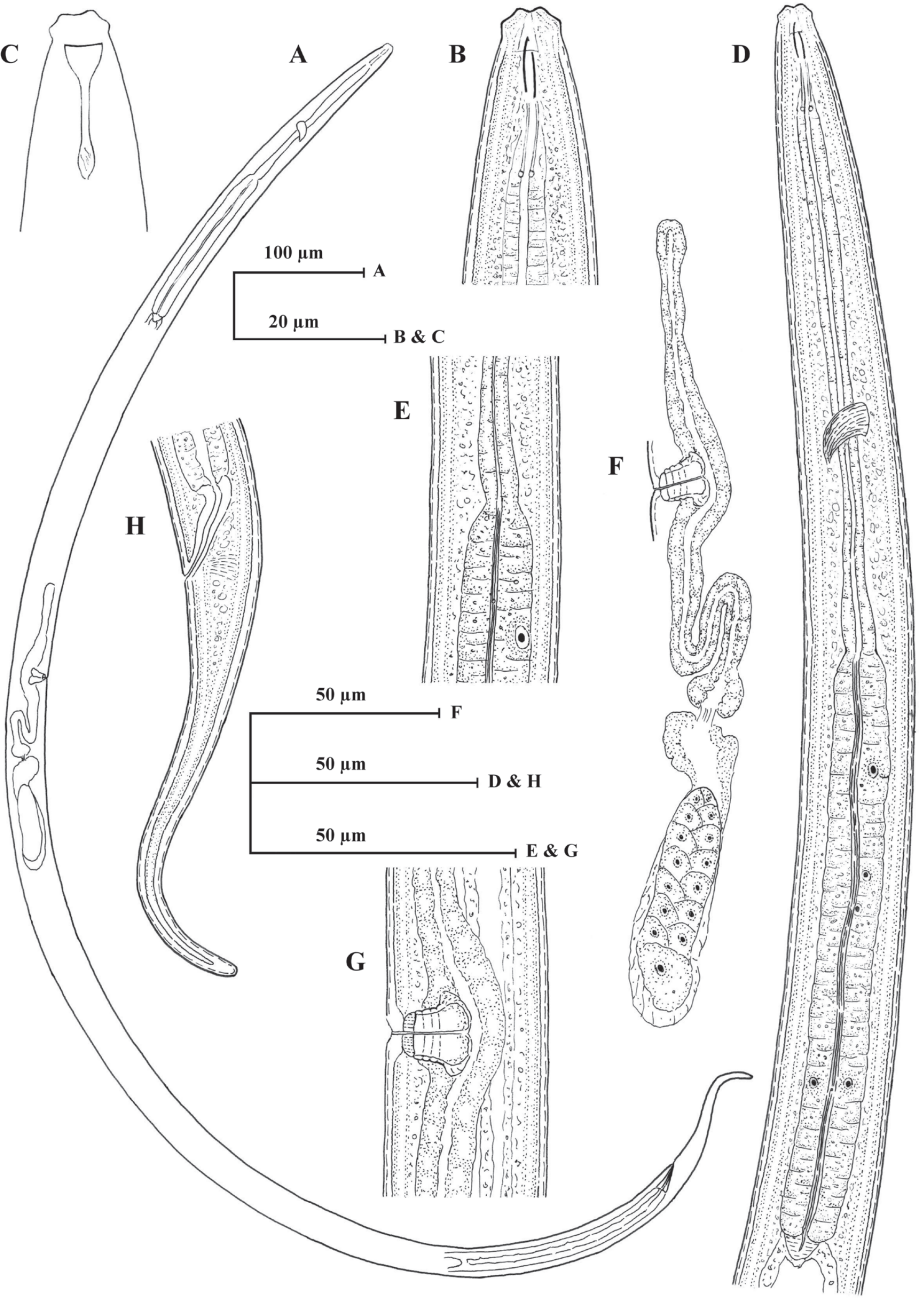
*Tahaminaindica* gen. nov., sp. nov. female: **A** entire **B** anterior region **C** anterior region showing amphid **D** pharyngeal region **E** pharyngeal expansion **F** genital system **G** Vulval region **H** posterior end.

**Figure 3.**
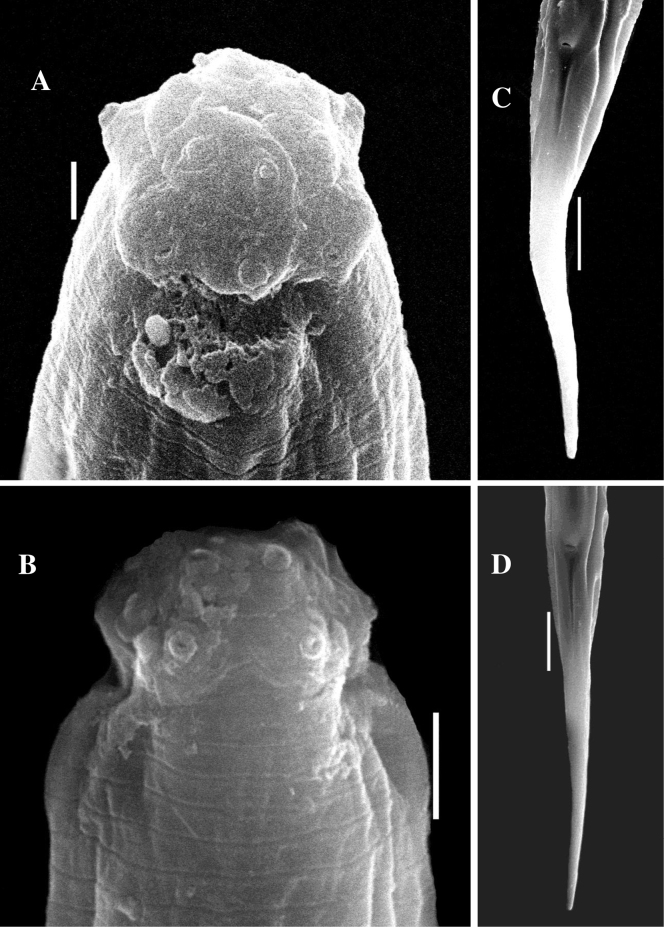
*Tahaminaindica* gen. nov., sp. nov. female (SEM): **A** anterior region frontal view **B** anterior region lateral view **C, D** posterior end showing anal opening. Scale bars: 1 µm (**A**); 2 µm (**B**); 10 µm (**C, D**).

**Figure 4.**
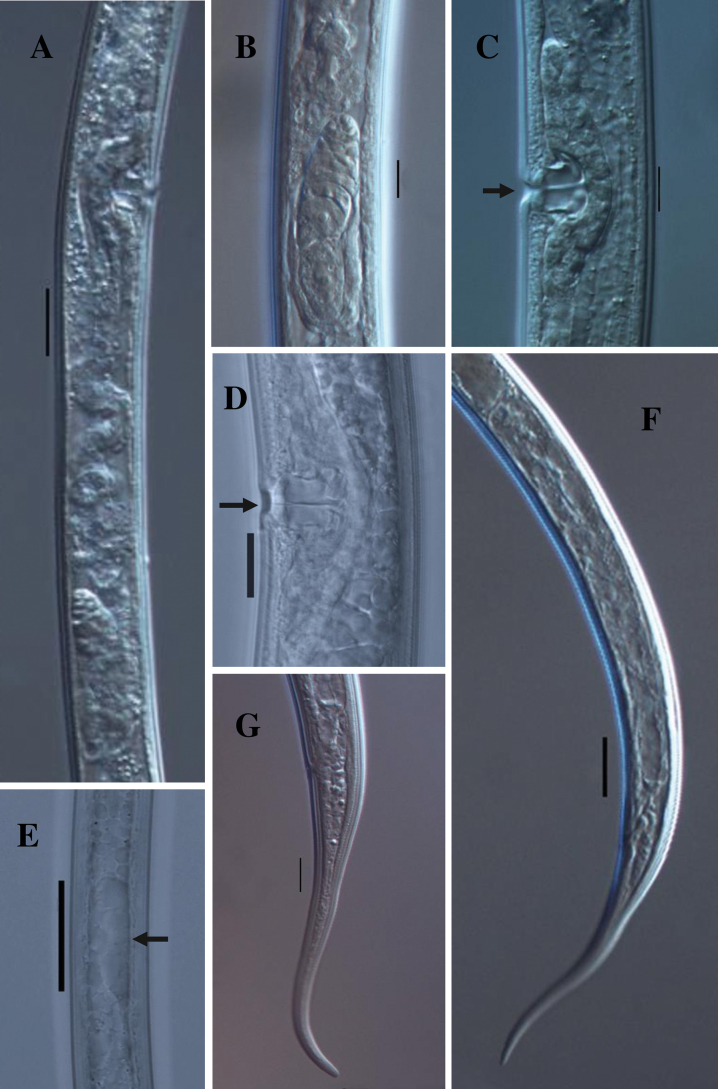
*Tahaminaindica* gen. nov., sp. nov. female: **A, B** genital system **C, D** vulval region **E** arrow showing genital primordia in juvenile **F** posterior region **G** posterior end. Scale bars: 20 µm (**A, F**); 10 µm (**B–D, E–G**).

Genital system mono-opistho-ovarian, didelphic. Ovary reflexed, measuring 50–78 μm long, not reaching oviduct-uterus junction; oocytes arranged in a single row except the near tip. Oviduct joining the ovary subterminally, measuring 57–82 μm, its consisting of a slender distal portion and a well-developed *pars dilatata*. Oviduct-uterus junction marked by the well-developed sphincter. Uterus long well differentiated, tripartite, proximal part short well-developed muscular, median part long, convoluted tube and distal part short, somewhat spheroid, measuring 96–109 μm. The anterior genital branch reduced to a simple sac, 34–69 μm or about 1.0–2.2 times midbody diameters long. Vagina cylindrical, extending inwards, 13.5–14.5 μm or about one-half (45–48%) midbody diameter; *pars proximalis vaginae* 10–12 × 6.0–8.0 μm, its wall encircled by circular muscles; *pars distalis vaginae* 2.5–3.5 μm with slightly curved walls; *pars refringens* absent. Vulva pore-like. Sperm cell absent. Prerectum long, 7.3–10.0 and rectum 1.1–1.4 anal body diameter long. Tail elongated, tapering gradually and its distal part slightly bent dorsally with a rounded terminus, 4.3–5.8 times anal body diameter long with a pair of caudal pores on each side.

**Male.** Not found.

**Juvenile (J2).** General morphology is similar to that of adults, but with the following differences. Medium sized nematodes, 1.0 mm long. Lip region cap-like, 2.1–2.3 times as broad as high or about one-third (29–34%) of body diameter at the pharyngeal base. Functional odontostyle 0.90 times the lip region diameter long, 0.80 times the replacement odontostyle length. Replacement odontostyle 1.0 times the lip region diameter long, with its base located at 45–48 μm from the anterior end. Position of odontostyle forming cell nucleus at 48–49 μm from the anterior end and at 26–28 μm from the odontophore base. Odontophore simple, rod-like, 1.5–1.6 times of functional odontostyle length. Guiding ring simple and refractive, at 0.7 times the lip region diameter from the anterior end. Pharyngeal expansion occupying 45–46% of the total pharyngeal length. Nerve ring at 33–34% of the total pharyngeal length from anterior end. Genital primordia 18–19 µm or 0.8–0.9 times the corresponding body diameter long, located at 442–445 μm or 41–43% of body length from the anterior end.

#### Type habitat.

Soil samples collected around the roots of grasses (*Cynodondactylon* L.) from Yaraganalu, Shivamoga District, Karnataka State, India.

#### Etymology.

The new genus and new species named after first author mother’s Tahamina and its type locality India.

## ﻿Discussion

In the present study, we have compared the new genus with some closely related genera either belong to Tylencholaimoidea (*Tylencholaimus*, *Heynsnema*, *Discomyctus*, *Wasimellu*s, *Dorylaimoides*) or Dorylaimoidea (*Mitoaxonchium*, *Hulqus*). The shape and size of the stylet, and the ratio of the expanded part of the pharynx of *Tahamina* displayed a close relation with some members of tylencholaimid nematodes. Similarly, based on especially the lip region and stylet morphology, we cannot ignore a comparison with two interesting members of the subfamily Hulqinae (*Mitoaxonchium*, *Hulqus*). Siddiqi (1981) proposed the subfamily Hulqinae under the family Discolaimidae (Dorylaimoidea) for the new genera *Hulqus* and *Mitoaxonchium* based on the length of the pharynx, more than one-third of body length (b = 2.4–3.2) and the position of dorsal pharyngeal gland nuclei at one-third length of expanded part of pharynx from its expansion. Although the new genus is comparable to members of Hulqinae based on the morphology of lip region and stylet, other morphology does not support its position under Hulqinae due to the presence of a shorter pharynx (b = 4.9–5.2) and the more anterior position of the dorsal pharyngeal gland nuclei (12–16 vs. 30% of expanded part of pharynx from its expansion). Furthermore, the cuticle of tylencholaimids is of two types: tylencholaimoid type; the inner layer usually thicker than the outer, loose, irregular outline, with distinct radial refractive elements (Tylencholaimidae and Leptonchidae) or dorylaimoid type; the inner layer of cuticle compacts, not loose, lack radial refractive elements but more or less distinctly striated (Tylencholaimellidae, Aulolaimoididae and Mydonomidae). Although, in the absence of radial refractive elements in our newly proposed genus, *Tahamina* is not supported under the family Tylencholaimidae, other morphological characteristics such as the shape and size of the stylet, the weakly muscular pharyngeal expansion, and the position of the dorsal esophageal gland nuclei well support the new genus under the subfamily Tylencholaiminae (Tylencholaimidae, Tylencholaimoidea).

## Supplementary Material

XML Treatment for Tahamina

XML Treatment for Tahamina indica

## ﻿Additional information

### Author ORCIDs

Md Niraul Islam https://orcid.org/0000-0001-8520-9775

### Data availability

All of the data that support the findings of this study are available in the main text or Supplementary Information.
